# Editorial: Recent Advances in Continuous Cultivation

**DOI:** 10.3389/fbioe.2021.641249

**Published:** 2021-02-22

**Authors:** Manfred Zinn, Thomas Egli, Christoph Herwig, Atul Narang

**Affiliations:** ^1^Institute of Life Technology, School of Engineering, University of Applied Sciences Western Switzerland, Sion, Switzerland; ^2^Department of Environmental Systems Science, Swiss Federal Institute of Technology, Zurich, Switzerland; ^3^Microbes-in-Water GmbH, Feldmeilen, Switzerland; ^4^Christian Doppler Laboratory for Mechanistic and Physiological Methods for Improved Bioprocesses, Vienna, Austria; ^5^Competence Center CHASE GmbH, Linz, Austria; ^6^Research Area Biochemical Engineering, Institute of Chemical Engineering, Vienna, Austria; ^7^Department of Biochemical Engineering and Biotechnology, Indian Institute of Technology Delhi, New Delhi, India

**Keywords:** chemostat, continuous culture, cell physiology, growth conditions, cultivation, growth medium, process analytical technology, growth kinetics

The scientific work by Monod (1950) and Novick and Szilard (1950a,b) on continuous cultivation in the 1950s and their early analyses and applications by the “Porton” and the “Prague” groups (Herbert et al., 1956; Malek et al., 1958; Pirt and Callow, 1960; Herbert, 1961) evened the path toward a better understanding of the cell physiology of microorganisms. The chemostat, where all physicochemical growth conditions can be kept constant, soon became a very important research tool to study the nutritional needs as a function of the specific growth rate of microorganisms. The recent evolution of new disciplines in systems biology offering a plethora of novel, scientific methods (e.g., transcriptional analysis, proteomics, metabolomics, and detailed metabolic flux analysis) played an important role in gaining a better understanding of the influence of the cell physiology and product formation.

To date, industry is investing in the development of continuous production processes, because of their better volumetric productivity and hence lower equipment cost. Moreover, powerful methods have been developed for continuous medium preparation and product purification (e.g., isolation of recombinant proteins in simulated moving bed chromatography). Finally, a continuous process can be run for an extended period and thus results in a more desired, ideally time invariant, and reproducible product quality. In future, more continuous bioprocesses will be used to produce pharmaceuticals, since the American Food and Drug Administration recently approved drugs produced by continuous cultivation (Matsunami et al., 2018). This change has been made possible because of the good experiences with their initiative Quality by Design (QbD) and the real-time control of cultivations using Process Analytical Technology (PAT).

The goal of this electronic article collection is to give an overview on recent scientific advances in continuous cultivation. In their review article, Nieto-Taype et al. summarize the potential of continuous cultivations of *Pichia pastoris* (*Komagataella phaffii*) with a particular focus on closed feed-back loop control using PAT. Typical examples are the turbidostat (cell density controlled), nutristat (nutrient concentration controlled), and among others, the pH-auxostat, where the substrate consumption changes the pH and thus triggers a pH changing substrate feed. In general, the cells cultured under these conditions are growing at almost maximum specific growth rate (μ_max_). Other interesting applications of continuous cultivation are changestats, where the dilution rate is continuously increased (accelerostats or A-stats), decreased (deceleration-stats or De-stats), or kept constant with a continuous change of another parameter (e.g., medium composition). A large potential of producing secondary metabolites and products is the retentostat, where all biomass is retained in a bioreactor and where the specific growth rate is practically zero.

Adamberg et al. applied the above described changestat approach to assess the adaptation of a human gut microbiota in A-stats and D-stats. The influence of the dilution rate (D) was tested either starting with steady-states at *D* = 0.05 h^−1^ or *D* = 0.2 h^−1^ and increasing or decreasing the dilution rate to *D* = 0.05 h^−1^ or *D* = 0.2 h^−1^, respectively. Interestingly, the detected population distribution was consistent and confirmed the fact that in continuous cultivation the Monod constant (Ks) plays a decisive role in the population dynamics. Finally, this finding is important to enable an industrial cultivation of multi-strain probiotics and fecal transplantation mixtures.

Barbera et al. describe the powerful approach of the chemostat cultivation to determine the necessary growth kinetics of the diazotrophic cyanobacterium *Anabaena* PCC7122 t model growth. The traditional batch approach was not appropriate, since along with the increase of the cell density, the average illumination is reduced and thus the metabolism is significantly affected. However, using small photobioreactors operated in a continuous mode, Barbera et al. were able to determine μ_max_ and the half saturation constant for nitrogen (K_N_). They also could show that the specific maintenance rate was influenced by the medium composition and the illumination intensity supplied to the culture.

Velu et al. investigated the potential of continuous outdoor cultures of the cyanobacterium *Tolypothrix* sp. by experimentally simulating the remediation of waste carbon dioxide (flue gas) and metal containing ash dam water from coal-fired power plants that are typically found in Australia. The entire process was economically feasible when the remediation was combined with the production of food-grade phycocyanin alone or in combination with biomass production as biofertilizer using either vertical or raceway suspension cultures.

The potential of the acetogenic bacterium *Clostridium autoethanogenum* to generate biofuel and chemicals from CO_2_ and H_2_ was assessed by Heffernan et al. using chemostat cultivations. The authors combined their experimental approach with a metabolic flux model and were thus able to optimize the conversion efficiency by supplementing the culture with CO. In future, this data set may be used to favor biosynthesis of a particular cell product.

Many physiological studies have shown that recombinant plasmids containing antibiotic resistance as selection marker may get lost without antibiotic selection, which renders this expression system failure prone and more expensive because of the need for continuous addition of antibiotics. However, new techniques, like CRISPR/CAS9 and related methods guarantee a better genetic stability. Nevertheless, the natural selection of better adapted cells (natural mutants; Novick and Szilard, 1950b) is leading to change in population distribution favoring the cells with a lower genetic burden. Because of such a population shift, the productivity may significantly be reduced or even lost completely. Kopp, Slouka et al. propose to evaluate the dynamic feeding and selection that takes place in the recently developed segregostat and also give an overview on existing online monitoring tools for *Escherichia coli*.

In industry, the product synthesis is frequently done in fed-batch cultivations in a phase separated manner, meaning the biomass formation is followed by the induction of genes of interest during the production phase. This set-up can also be established in a continuous manner by using two-stage chemostats. Kopp, Kolkmann et al. exemplified such recombinant protein production (RPP) in *Escherichia coli* in a two-stage chemostat using glycerol as a carbon source in the first stage and glycerol and lactose as inducers replacing IPTG in the second stage. The space-time-yield could be increased by a factor of 100 in comparison to a single-stage chemostat cultivation.

This continuous two-stage cultivation technique has also been implemented in research studies. Hanik et al. reported the production of tailor-made medium chain length poly(3-hydroxyalkanoate) (mcl-PHA) in two-stage, parallel chemostat cultivations. In the first stage (one bioreactor) *Pseudomonas putida* was grown and in the second chemostat stage consisting of 4 parallel chemostats the substrates for mcl-PHA production (phenylvaleric and decanoic acids) were added. This approach allowed a faster identification of the design window for tailor-made PHA production.

Tapia et al. developed a two-stage chemostat cultivation technique in order to assess the viral dynamics and the interaction of defective interfering particles (DIPs) of influenza A virus with the canine cell line MDCK.SU2. The design of a mathematical model enabled the interpretation of the observed cell and viral oscillations in the second chemostat stage. It was concluded that continuous two-stage cultivations could be efficiently used to produce DIPs.

## Author Contributions

MZ has drafted the manuscript and submitted in final form. TE, CH, and AN have revised the manuscript draft. All authors contributed to the article and approved the submitted version.

## Conflict of Interest

The authors declare that the research was conducted in the absence of any commercial or financial relationships that could be construed as a potential conflict of interest.
